# Evaluating the Efficacy of Pervistop^®^, a New Combination Based on EGCG, Folic Acid, Vitamin B12 and Hyaluronic Acid on Patients with Human Papilloma Virus (HPV) Persistent Infections and Cervical Lesions: A Pilot Study

**DOI:** 10.3390/jcm12062171

**Published:** 2023-03-10

**Authors:** Cesare Aragona, Maria Salomè Bezerra Espinola, Gabriele Bilotta, Giuseppina Porcaro, Marco Calcagno

**Affiliations:** 1 Systems Biology Group Lab, 00161 Rome, Italy; 2 Alma Res Fertility Center, 00198 Rome, Italy; 3 Women’s Health Centre, USL UMBRIA 2, 05100 Terni, Italy; 4 Department of Woman Health and Reproductive Medicine, Santo Spirito Hospital, 00193 Rome, Italy

**Keywords:** HPV, persistence, ASCUS, LSIL, EGCG, folic acid, vitamin B12, hyaluronic acid

## Abstract

Background: The persistence of the HPV infection is a risk factor in the integration of viral DNA in the host genome, leading to transforming events. The lack of therapies for HPV-persistent infections determine an unmet medical need. Methods: We enrolled forty patients with persistent HPV infections and cervical lesions and divided them into two groups. The women in the treated group received 200 mg epigallocatechin gallate (EGCG), 400 µg folic acid (FA), 1 mg vitamin B12, and 50 mg hyaluronic acid (HA) for 12 weeks. The control group received no treatment. Results: 40 patients completed the study. Fifteen out of 20 women in the control group still had an LSIL at the end of the study. One woman had a decrease in the DNA load, while six had no change and eight had an increase in DNA content. In the treatment group, 17 out of 20 women achieved a full viral clearance. These women showed no cytological or histological evidence of lesions following the treatment. Conclusions: Our data highlight the possible effect of such combination on LSIL. Therefore, the evidence reported here supports the potential to carry out further randomized placebo-controlled studies with an adequate number of patients to verify our results.

## 1. Introduction

Human papilloma virus (HPV) is a family of double-strand DNA virus that can be transmitted through various types of contact, including skin-to-skin, skin-to-mucosa, and mucosa-to-mucosa. HPVs are non-enveloped viruses that generally penetrate the cells via a clathrin-dependent mechanism. A feature of papilloma viruses is the lack of coding regions for any DNA polymerase in their genomes. In order to replicate by taking advantage of the host’s polymerase, the target tissues of these viruses are those characterized by active and continuous replication. Therefore, HPV is a family of viruses that affect skin and mucosa, particularly genital, oral, and anal. HPV infections are diagnosed especially in women, and the estimates suggest that 80% of sexually active women contract this infection during their lifetime [1].

In the greater part of the cases, HPV infections resolve spontaneously, without any relevant clinical manifestation. In the case of clinical manifestations, HPV is responsible, in most cases, for benign lesions such as skin warts or condylomas, the latter occurring in the affected mucosa. These benign lesions usually heal spontaneously although in some cases they may require treatment, usually consisting of ointments or minor procedures. More rarely, HPV infections affecting the mucosa can give rise to malignant lesions and evolve into cancers. Indeed, HPVs are classified based on their risk of transforming healthy cervical tissue into a cancerous mass. The most accurate classifications identify two levels of risk, determining to a classification divided in high-risk strains and low-risk strains [2]. 

Among the possible HPV-induced cancers, the most burdensome for women’s health globally is cervical cancer. Cervical cancer is the 4th most common cancer worldwide, with 604,127 new cases and 341,831 deaths in 2020. Considering the female population, cervical cancer is the 2nd most common cancer in the world in women aged between 15 and 44 years [3]. Mortality is affected by the stage, time of diagnosis, and the accessibility and efficacy of treatments. In 2020, cervical cancer represented the 5th most frequent cancer in Italy in women under the age of 50, with 2400 new cases, representing 1.3% of all cancers affecting the female population [4,5]. 

The persistence of the infection has different definitions. Generally, persistence is defined as the detection of the same virus strain affecting a patient in two temporally distant moments. The time interval between the two tests shifts from nine months to two years [6]. The persistence phenomenon is important as it is a risk factor in the integration of viral DNA in the host genome [6]. The integration of the genome is the real risk factor for the occurrence of transformation of the epithelium: the virus strains with the greatest oncogenic risk (HR-HPV), in particular HPV-16 and -18, are those that have the greatest persistence [7]. As a viruses persistence mainly depends on viral DNA integration into the cellular genome, and on the properties of the different viral strains to evade the immune response of the host, HPV-16 and -18 are the strains that expose the patients to a higher transforming risk. More precisely, the virus strains that have the greater persistence have the higher chance to integrate their DNA in the host’s genome, thus exposing patients to the higher risk of neoplastic transformation. In the case of high-risk viral strains as HPV-16 and -18, precancerous lesions have the higher chance to progress into cervical cancer due to the increased chance of integration [8,9,10,11]. Once the virus has integrated its DNA in the host’s genome, the expression of the viral gene E2 terminates. Indeed, E2 is a protein expressed by the virus that blocks the transcription of viral genes E6 and E7. Therefore, once integrated, the block on the expression of E6 and E7 is removed, allowing the transcription of such genes. These genes are indeed suppressors of p53 and pRB, which are both host proteins that prevent the cells from degenerating into a cancerous mass by inducing apoptosis. Therefore, the expression of E6 and E7 reduce the host control of the cells’ apoptosis [12]. 

According to the WHO histopathological classification system, cervical intraepithelial neoplasms (CIN) are characterized by alterations such as mild dysplasia (CIN1), moderate dysplasia (CIN2), severe dysplasia (CIN3), and carcinoma in situ (CIS) [13]. A revised cytological nomenclature system known as “The Bethesda System” classifies squamous intraepithelial lesions as low-grade (low-grade squamous intraepithelial lesion, LSIL), generally matching CIN1, and high-grade lesions (high-grade squamous intraepithelial lesion, HSIL), which generally refers to lesions classified as CIN2 and CIN3. Cytological results classified as atypical squamous cells (ASC) are divided in “of undetermined significance “(ASC-US) or as “cannot exclude HSIL” (ASC-H) [14,15]. 

Despite numerous advances in HPV research and screening campaigns with Pap smear and HPV-DNA test, HPV-persistent infections have no specific treatments, an issue that remains to be solved. In this perspective, developing new therapeutic strategies to act against the persistence of HPV infection is crucial. 

Several non-pharmacological molecules are increasingly gaining importance in the treatment of a plethora of tumors, including those induced by papilloma virus [16,17,18,19]. Among those molecules, polyphenols contained in green tea leaves display biochemical properties such as the ability to inhibit the onset and the spread of tumors. Indeed, polyphenols are powerful antioxidants, able to neutralize free radicals, thus preventing cell damage. Therefore, green tea extract has been largely studied and employed in several scientific research studies. Green tea polyphenols are composed of six types of catechins and their derivatives (gallates), and epigallocatechin gallate (EGCG) is considered the substance with the stronger antiproliferative effects among them [20]. In the context of HPV infections, this molecule exerts its action by blocking cell growth during virus persistence. Indeed, EGCG induces p53 expression while impairing the expression of E6/E7 viral oncoproteins, thus inducing apoptosis in HPV-infected cells. Finally, EGCG presents chemo-preventive action on HPV lesions, inducing the disappearance of condylomas (both in males and females) [21,22,23,24,25]. 

Tumors are multifactorial diseases, relatable to environmental, food, genetic and epigenetic factors. Indeed, the lack of several nutrients may contribute to cancer development. Among them, literature reports the crucial role of folic acid (FA) and vitamin B12 in HPV-positive patients [26,27]. 

FA and vitamin B12 can prevent HPV proliferation by controlling DNA methylation. In particular, such methylation prevents the transcription of several genes, including those involved in virus persistence [28]. 

In the context of HPV infection, hyaluronic acid (HA) is crucial to repair lesions. HA is essential to activate several pro-inflammatory factors that allow wound healing [29]. HA accelerates the healing process and restores the integrity of the epithelium and mucous membranes, preventing entrance in cells of viral particles, thus impairing infection process [29,30,31]. 

In this study, we aimed to test the efficacy of the association of EGCG, FA, vitamin B12, and HA in patients with persistent HPV infections and cervical lesions.

## 2. Materials and Methods

This study was a pilot, open label, controlled clinical trial. Participants were recruited at Clinica ALMA RES from June 2022 to August 2022. The study was approved by the Institutional Review Board of Clinica ALMA RES (Ref. No. 014/2022) and conducted according to the ethical principles of the Declaration of Helsinki. The trial was registered on ClinicalTrials.gov (Ref. No. NCT05625308). Following Institutional Review Board approval, patients were asked to sign an informed consent explaining the purpose of the study and the study protocol. The inclusion criteria were (1) females aged 30 to 45 years, (2) HPV-persistent infection for more than two years before entering the study, defined as positivity to the same HPV genotype in two different HPV-DNA tests performed at least two years apart, (3) PAP test indicating LSIL or ASC-US cytology, and (4) colposcopy and biopsy confirming LSIL. The exclusion criteria included (1) cervical cancer patients, (2) diagnosis of HSIL, (3) previous surgical procedures for HPV-induced transformation (e.g., conization or loop electrosurgical excision procedure), (4) concurrent uterine pathologies, and (5) pregnancy or intended to seek pregnancy in the next three months. 

All women received cervical smears, HPV-DNA test, and colposcopy with biopsy to confirm the HPV infection. The primary outcome of the study was the clearance from the viral infection, which is inversely related to virus persistence rate. Therefore, the main outcome was the rate of negative responses to the HPV-DNA test in patients with a history of positivity to HPV-DNA test and LSIL longer than two years. The secondary outcomes were negative response to PAP test and to biopsy analyses. As the combination of EGCG, HA, FA, and vitamin B12 had never been tested before in a clinical trial, we could not perform a power analyses to determine a sample size. Therefore, we could not select a sample size to be enrolled for our pilot study. Patients who wished to enter in the treatment group received a box containing all the tablets for a 12-week treatment with one tablet per day via oral route. Conversely, patients who decided to enter in the study as control underwent no treatment but followed a regimen of clinical surveillance as suggested by the Italian guidelines. Tablets given to the patients in the treatment group consisted of a combination of EGCG 200 mg, HA 50 mg, FA 400 μg, and vitamin B12 1 mg (Pervistop^®^, Farmares, Italy). After three months from the study start, all the patients included in the study underwent a new complete medical examination, including PAP test, HPV-DNA test, and colposcopy. Moreover, in the case of positivity to colposcopy, we performed a biopsy to examine the exact extent of the lesion. 

We also evaluated side effects following the treatment to establish the safety of the tested combination of molecules. Over the time of the treatment we evaluated the incidence of the following symptoms: dizziness, confusion, cold sweat, weakness or discomfort, and gastrointestinal disturbs and vaginal disturbs (namely vaginal burning, itching, or inflammation). Patients were also encouraged to report any other side effects.

During PAP test, the swab was transferred to a glass microscope slide, fixed in 95% ethanol, and treated using the Papanicolaou′s solution 2a orange G (Sigma-Aldritch, Saint Louis, MI, USA). The same anatomical pathologist examined all the glasses and classified the results according to the Bethesda System. For HPV-DNA testing, we used Hybrid Capture 2 (HC2) test kits (Digene, Silver Spring, MD, USA) according to the manufacturer’s instructions. We considered samples with a relative light unit (RLU) higher than 1 as positive to HPV-DNA test. We compared age, height, weight, Body-Mass Index (BMI), parity, and time of virus persistence between the two groups at baseline to obtain statistically matching samples of patients. We analyzed data through student’s T-test for parametric distribution of continuous data. We analyzed outcomes with a dichotomous variable through chi square test. All data were analyzed using GraphPad Prism Software. We considered statistically significant a *p* value < 0.05.

## 3. Results

We assessed a total of 57 women for eligibility. Twelve were excluded as they did not match our inclusion criteria or matched the exclusion criteria. Four women declined to participate, and the remaining 41 women with HPV infection were enrolled and asked about their preferences to enter in the intervention (20 women) or in the control (21 women) groups. No patient was further excluded; however, we had a dropout from the study as a patient in the control group was lost to follow-up. No patient in the treatment group reported an interruption of the treatment. Table 1 summarized patients’ data concerning age, weight, height, BMI, parity, and time of virus persistence. Concerning these data, we found no significant differences between the intervention and control groups both at the baseline and following the treatment. In the control group, 11 women had HPV-18 genotype, seven women had HPV-16 genotype, one had HPV-33, and the latter had HPV-52. In the treatment group, 10 women had HPV-18, five had HPV-16, two had HPV-45, two had HPV-33, and one had HPV-52. 

With regards to viral DNA presence, HPV-DNA test revealed a clearance rate following the twelve week period equal to 85.0% and 25.0% in the intervention and in the control group, respectively (Table 2). Chi square test revealed a significantly higher clearance in the intervention group with respect to the control group (*p* = 0.000137). 

Results of the PAP test, HPV-DNA test, and biopsy before and after the treatment or the 12-week period are presented in Table 3. We found significant differences in the results of PAP test and of biopsy between the two groups following the 12-week interval. The treatment induced a significant decrease in the abnormal PAP smear rate in the study group (20 to 3) compared to the control group (20 to 15). The specimen obtained through biopsies matched the results of the PAP test and the HPV-DNA test: the biopsies positive for lesions fell from 20 to 3 in the intervention group and from 20 to 15 in the control group. 

By analyzing the results in relation to the genotype, we could calculate the clearance rates among the different genotypes. Among the 17 women in the treatment group that proved to have a viral clearance, eight women had HPV-18, four had HPV-16, two had HPV-33, two had HPV-45, and one had HPV-52. Therefore, the clearance rate was 80% in the case of HPV-18 (eight patients out of 10) and 80% in the case of HPV-16 (four patients out of 5).

Concerning the side effects, patients under treatment with 200 mg of EGCG, 400 mcg of folic acid, 1 mg of vitamin B12 and 50 mg of hyaluronic acid for 12 weeks reported minor or null side effects. Only one patient reported the occurrence of gastrointestinal disturbs during the first 4 weeks of treatment. Patients reported no other adverse events. 

## 4. Discussion

To the best of our knowledge, this is the first clinical study investigating the role of EGCG, FA, HA, and vitamin B12 in papillomavirus-persistent infection. Moreover, this study provides the first insight into the therapeutical activities of natural compounds in such clinical picture. Indeed, we found out that an oral treatment with tablets containing 200 mg of EGCG, 400 mcg of FA, 1 mg of vitamin B12, and 50 mg of HA for 12 weeks reduces the rate of viral persistence. Despite our clinical trial providing the first evidence on such combination, the effects of EGCG alone, folic acid alone, hyaluronic acid alone, and vitamin B12 alone are already described in the literature [32,33,34,35,36,37]. 

Zou and colleagues [21] carried out an in vitro study to determine the effect of EGCG in cancer cell lines positive to HPV infection. Indeed, their results demonstrated that EGCG treatment induces apoptosis in cancer cell lines infected with HPV-16 and -18 strains in a dose-dependent manner. Moreover, in cancer cells, EGCG promotes the expression of two crucial pro-apoptotic factors: p53 and p21. The mechanism by which EGCG reverses the inhibition of the pro-apoptotic factors depends on the effect of EGCG on viral oncoprotein E7. Indeed, EGCG treatment reduces the expression of E7 viral protein, thus dampening virus persistence and promoting the apoptosis in the infected cells. Further investigations on the relationship between EGCG treatment and viral oncoproteins revealed the wide effect of such molecules [22]. 

Another study proved that EGCG treatment reduces the expression of both E6 and E7. This finding is pivotal to understand the efficacy of EGCG in reducing the malignant transformation rate of cervical lesions in HPV positive patients, as E6 and E7 increasingly abolish the control of the cells on their own apoptotic processes. Therefore, by reducing E6 and E7 expression, EGCG alone is able to induce a slight reduction in the chance of viral persistence. Moreover, there is weak evidence on the ability of EGCG to prevent viral DNA inclusion in the human genome, but this evidence still needs to be further validated [8]. 

In another in vitro study, the authors treated two HPV18-positive cell lines derived from foreskin keratinocytes and vulvar intraepithelial neoplasia with EGCG for 24, 48, and 72 h. Their results indicate that EGCG treatment significantly downregulates E6 and E7 viral proteins, promoting the degradation of both the proteins through the proteasome. Moreover, such reduction in the expression of viral oncogenes increases the expression of pro-apoptotic factor p53 and pRB. Consequently, the proliferation of the keratinocytes and the vulvar neoplasia cells decreased, restoring a near-healthy phenotype. Moreover, EGCG displayed an important inhibitory activity on E4 expression. E4 is the viral protein responsible for viral DNA replication; therefore, EGCG treatment also blocks the replication of the virus, contributing to reduce the extent of the infection [25]. 

From a clinical point of view, a study involving 90 women with different-graded cervical lesions revealed that EGCG oral treatment has chemo-preventive effects in vivo. The authors prescribed a treatment consisting of an oral tablet containing 200 mg of EGCG daily, examining the response to the treatment, considered as the histological absence of lesions. The results report an improvement in 60% of the cases following oral treatment; in addition, the authors report a positive response in 71% of the patients with severe dysplasia, namely HSIL [23]. 

A following study on the efficiency of EGCG involved 226 men and 227 women with anogenital condyloma. The authors prescribed a treatment with topical applications of an ointment consisting of green tea extracts and containing a variable amount of EGCG (50–72%). Following the application of the ointment three times per day for 16 weeks, the authors examined the complete clearance rate following the treatment. They report that 53% of the cases had a total disappearance of the lesions after EGCG treatment. Moreover, they state that the treatment is far more efficient in women than in men, with a complete clearance rate equal to 60% of the cases in women and 45% of the cases in men. In addition, they report that 78% of patients treated with EGCG ointment had a clearance equal to or higher than 50%. The authors also state that the effects of EGCG on the regression of condylomas derives from its block on cell proliferation, inducing apoptosis and leading to a regression or total disappearance [24]. 

FA and vitamin B12 also displayed important properties in the case of HPV infection. Piyathilake et al. [27] carried out a study involving 315 women to evaluate the correlation between HPV genome methylation and FA and vitamin B12 levels. Their results highlighted that high levels of FA and vitamin B12 are related to high viral genome methylation. This eventually leads to a minor transcription of viral genome, halting the replication of the virus and the formation of new virions. Indeed, the authors highlighted that, due to these mechanisms, high levels of FA and vitamin B12 correlated with lower grades of the lesions. On the contrary, women with reduced levels of FA and vitamin B12 displayed higher-grade injuries. 

Sedjo et al. [28] examined the correlation between the levels of different micronutrients and the persistence of HPV infection. Their results provided a confirmation of the vitamin B12 effect in HPV-positive patients. Indeed, the authors highlighted that circulating levels of vitamin B12 in HPV-positive women are inversely correlated with HPV persistence. Moreover, they pointed out that women with higher serum levels of vitamin B12 were less likely to have viral persistence and were, therefore, at a lower risk of developing high-grade lesions and carcinoma. In another study, Abike et al. [36] investigated the relationship between HPV-infection persistence and serum levels of micronutrients, including folate and homocysteine. They highlighted that folate plasma levels inversely correlates with the occurrence of lesions. This means that women with ASC-US, LSIL, or HSIL had lower levels of circulating folate with respect to healthy controls. They also analyzed the plasma levels of homocysteine in women with or without HPV-induced lesions. Indeed, homocysteine can be considered as an indirect measure of folate and vitamin B12, as the latter two participate in the homocysteine catabolism, promoting the conversion of homocysteine to methionine. Therefore, high plasma levels of homocysteine imply lower levels or activity of folate and vitamin B12. According to their other results, serum homocysteine levels were higher in the group of women with HPV-induced lesions. Moreover, they suggested that the folate deficiency can be considered as a risk factor predisposing for HPV-infection persistence and progression of cervical dysplasia. Intriguingly, the results by Abike et al. are complementary to those obtained by Sedjo et al., as the two groups correlated the progression of HPV infection to the levels of folate and vitamin B12. Yenigul et al. [38] carried out a following study on the correlation between the presence of ASC-US and HPV persistence with folate and vitamin B12 serum levels. Indeed, they analyzed the blood levels of 200 patients divided in ASC-US cytology and normal cytology groups. Their results highlighted that patients positive to HPV-DNA test with ASC-US cytology had lower serum levels of folate and of vitamin B12 with respect to patients without ASC-US or with ASC-US but negative to HPV-DNA test. Taken together, the results from the aforementioned groups clearly indicate that folate and vitamin B12 may represent useful integrative therapies in the case of HPV-persistent infection, presumably by involving HPV genome methylation. 

Hyaluronic acid is particularly important in the case of HPV infections for tissue repair and homeostasis. HA plays an important role in restoring the integrity of epitheliums and mucous membranes. In the case of HPV-positive patients, due to its effects in maintaining the integrity of mucosa, HA is effective in preventing the entry of viral particles into the cells, blocking the infection process. Riemma et al. [37] also provided the first evidence on the clinical effects of HA in HPV-infected women with LSIL. Indeed, they tested a combination of natural products in presence or absence of HA topical treatment. Their results showed greater benefits in the presence of HA, suggesting an adjuvant effect for this molecule in the case of HPV infections. We hypothesize that on the one hand the formula has the main activity in counteracting the progression of the HPV-persistent infection and of the HPV-induced lesions, while HA exerts repairing effects. As HPV penetrates the abrasions in the mucosa to reach the deeper layer of stem cells, a successful strategy to prevent virus persistence may comprehend the promotion of the regeneration of tissues. Therefore, by promoting the wound healing, HA may be useful in avoiding viral persistence and preventing the recurrence of viral infection [30]. 

Hyaluronic acid is a well-known moisturizer, and literature highlights that its effects may differ based on the molecular weight of the molecules. Indeed, HA is a chain composed of hundreds of condensed disaccharides of glucuronic acid and N-acetyl-glucosamine. Therefore, the activities of such polymers may differ depending on the number of repetitions. Indeed, literature highlighted that high-molecular-weight hyaluronic acid (weighting more than 500 kDa) has anti-inflammatory properties, while low-molecular weight hyaluronic acid (weighting less than 500 kDa) has lubricant properties. Indeed, the HA that was comprised in the formula that we tested is called very low-molecular weight hyaluronic acid, which has a molecular weight lower than 10 kDa. Literature evidence supports the use of very-low molecular weight HA in the case of wounds or abrasions. This is based on the evidence on the molecular activities of a particular type of HA, which is known to deeply penetrate into the wounds and abrasions, filling the injured tissues that HPV would penetrate; thus, promoting the repair [29]. 

Our study aimed to test the efficacy of the combination of EGCG, FA, vitamin B12, and HA. Our results confirmed the evidence on the single molecules, also providing information about the potential efficacy of such combination against HPV-persistent infections. Our data suggest that the oral supplementation with such combination for 12 weeks leads to a positive response in HPV-positive women with viral persistence. From a cytological point of view, seven out of eight patients with ASC-US and 10 out of 12 patients with LSIL displayed positive responses after treatment. From a histological point of view, 17 out of 20 patients with LSIL displayed a negative response to the biopsy following the treatment. Therefore, our data also suggest that the combination of EGCG, FA, vitamin B12, and HA may represent a promising treatment for suppressing HPV persistence. Interestingly, we obtained matching results with PAP tests, HPV-DNA tests and biopsies, suggesting an eradication of the viral infection and a matching positive effect on the lesions. We also stratified the results based on the HPV genotypes; however, we could not include a significance analysis due to the small sample size representing the different genotypes. Nonetheless, when considering the overall response rate, our data indicate that such treatment with the combination of EGCG, FA, vitamin B12, and HA can be considered as an effective therapy, as patients reported no side effects.

Our results provide evidence on a new possible treatment for HPV-persistent infection. However, this study involves only a small number of patients; therefore, our result cannot confirm the efficacy of the treatment. Based on the evidence we produced, we hope to prompt research on these molecules in treating women affected by LSIL. As this is a pilot study involving a small sample size, we strongly encourage further double-blind, randomized, and placebo-controlled studies to verify our preliminary results.

## 5. Conclusions

Although the population size of this study is small, our clinical observation provides the first evidence on the combination of EGCG, FA, vitamin B12, and HA in treating LSIL. Moreover, our observations indicate that such combination of natural molecules may represent a promising treatment for women with persistent HPV infection. Despite our promising results, our findings should be verified in larger cohort studies with an appropriate randomization plan and a placebo treatment as control. Based on our findings, further studies may involve an adequate number of patients to confirm with certainty our results. In summary, our study suggests that a combination of EGCG, FA, vitamin B12, and HA may exert preventive effects against the progression from mild dysplasia to a more severe stage of cervical neoplasia. However, more structured studies are needed to verify our preliminary results.

## Figures and Tables

**Table 1:** Baseline characteristics of patients in the study.

Characteristics	Control Group	Treatment Group	*p*-Value
Age (years)	37.65 ± 2.48	37.35 ± 2.60	0.71
Weight (kg)	68.03 ± 7.15	68.57 ± 7.46	0.83
Height (m)	1.65 ± 0.06	1.66 ± 0.06	0.85
Body-Mass Index (kg/m^2^)	24.88 ± 2.52	24.92 ± 2.15	0.96
Parity (n°)	0.85 ± 0.74	0.95 ± 0.94	0.71
Viral persistence (years)	3.10 ± 0.67	3.04 ± 0.66	0.81
Smokers (%)	2 (10%)	3 (15%)	0.63
Immunodeficient (%)	0 (0%)	0 (0%)	1

**Table 2:** Changes in HPV-DNA content of cervical pre-cancerous lesions by treatment with a tablet per day containing 200 mg of EGCG, 400 mcg of FA, 1 mg of vitamin B12, and 50 mg of hyaluronic acid for 12 weeks.

Group	Post-Treatment Effects on DNA Content (no. Patients)			
	Disappear	Decrease in DNA Content	No Change	Increase in DNA Content
Treated (n = 20)	17 (85%)	2 (10%)	1 (5%)	0 (0%)
Control (n = 20)	5 (25%)	1 (5%)	6 (30%)	8 (40%)

**Table 3:** Overall response rates after 12 weeks of the treatment versus natural histology of HPV-induced lesions as control. PAP Test: Papanicolau Test; HPV: Human Papilloma Virus; ASC-US: Atypical Squamous Cells of Undetermined Significance; LSIL: Low-grade Squamous Intraepithelial Lesion.

Group	PAP Test		HPV-DNA Test		Biopsy	
	Positive	Negative	Positive	Negative	Positive	Negative
Treated (n = 20)	3 (15%) (2 ASC-US, 1 LSIL)	17 (85%)	3 (15%)	17 (85%)	3 (15%) (3 LSIL)	17 (85%)
Control (n = 20)	15 (75%) (3 ASC-US, 12 LSIL)	5 (25%)	15 (75%)	5 (25%)	15 (75%) (15 LSIL)	5 (25%)
*p*-value	0.000137		0.000137		0.000137	

## Data Availability

Data are available from the corresponding author on a reasonable request.
